# Lysozyme alleviates DSS-induced colitis by modulating pro-inflammatory factors and NF-κB activation

**DOI:** 10.1093/lifemedi/lnaf020

**Published:** 2025-06-14

**Authors:** Kai Wang, Lan Guo, Ting Wang, Fengmin Shi, Meihong Fu, Guojun Li, Songli Pan, Xianying Cao, Huanxiong Chen

**Affiliations:** Department of Spine Surgery, the First Affiliated Hospital of Hainan Medical University, International Center for Aging and Cancer, Hainan Academy of Medical Sciences, Hainan Medical University, Haikou 571199, China; College of Food Science and Technology, State Key Laboratory of Marine Resources Utilization of South China Sea, Hainan University, Haikou 570228, China; College of Food Science and Technology, State Key Laboratory of Marine Resources Utilization of South China Sea, Hainan University, Haikou 570228, China; Shanxi Bethune Hospital, Shanxi Academy of Medical Sciences, Tongji Shanxi Hospital, Third Hospital of Shanxi Medical University, Taiyuan 030032, China; Department of Spine Surgery, the First Affiliated Hospital of Hainan Medical University, International Center for Aging and Cancer, Hainan Academy of Medical Sciences, Hainan Medical University, Haikou 571199, China; Department of Spine Surgery, the First Affiliated Hospital of Hainan Medical University, International Center for Aging and Cancer, Hainan Academy of Medical Sciences, Hainan Medical University, Haikou 571199, China; Department of Spine Surgery, the First Affiliated Hospital of Hainan Medical University, International Center for Aging and Cancer, Hainan Academy of Medical Sciences, Hainan Medical University, Haikou 571199, China; Department of Spine Surgery, the First Affiliated Hospital of Hainan Medical University, International Center for Aging and Cancer, Hainan Academy of Medical Sciences, Hainan Medical University, Haikou 571199, China; College of Food Science and Technology, State Key Laboratory of Marine Resources Utilization of South China Sea, Hainan University, Haikou 570228, China; Department of Spine Surgery, Hainan Province Clinical Medical Center, the Second Affiliated Hospital of Hainan Medical University, Hainan Academy of Medical Sciences, Hainan Medical University, Haikou 570311, China

## Dear Editor,

Ulcerative colitis (UC) is a chronic, idiopathic, and recurrent inflammatory disease affecting the mucosal of the rectum and colon. No sex predominance has been observed, and the peak incidence occurs between the ages of 30 and 40 years old [1, 2]. In recent years, many studies have shown that the prevalence and incidence of UC have continued to increase worldwide. Although the pathogenesis of UC remains unclear, increasing evidence suggests that defects in the epithelial barrier and imbalances in the intestinal mucosal immune system contribute to disease progression [3, 4]. Patients with UC exhibit reduced colonic goblet cell counts and increased intestinal mucosal permeability, indicating that defects in barrier function are a primary driver of the disease [3]. Immune imbalance in the intestinal mucosa leads to the excessive production of pro-inflammatory factors, such as TNF-α and IL-1β, which perpetuate inflammation and exacerbate colon damage [5]. Furthermore, the upregulation of inflammatory mediators, including COX-2 and iNOS, may contribute to persistent inflammatory responses of the intestinal mucosa. Currently, sulfasalazines, glucocorticoids, and certain biological inhibitors are commonly used in the treatment of UC [4]. However, these therapies are associated with significant immediate side effects and long-term toxicity. Consequently, the use of natural ingredients derived from food, which possess health benefits, has increasingly attracted the interest of researchers in medical treatment.

Lysozyme (Lys) is an antimicrobial protein found in egg whites. Peptides derived from Lys can modulate innate immunity by binding to DNA molecules, interfering with DNA replication, and altering gene expression [6, 7]. Furthermore, dipeptides, tripeptide, and their derivatives are transported into the cytosol in the intestine via the oligopeptide transporter (PepT1) to exert anti-inflammatory effects [8]. Notably, this receptor is expressed in colonic epithelial cells of chronic UC but not in normal colonic epithelial cells. Recent studies suggest that Lys ameliorates dextran sodium sulfate (DSS)-induced colitis by restoring mucosal immune homeostasis, downregulating local gene expression, suppressing pro-inflammatory factors, and enhancing the intestinal mucosal chemical barrier [9]. However, the protective mechanism of Lys in UC remains unclear. This study demonstrates that Lys exerts anti-inflammatory effects in the colonic mucosa under acute conditions by regulating the expression of transcription and pro-inflammatory mediators.

**Figure 1.**
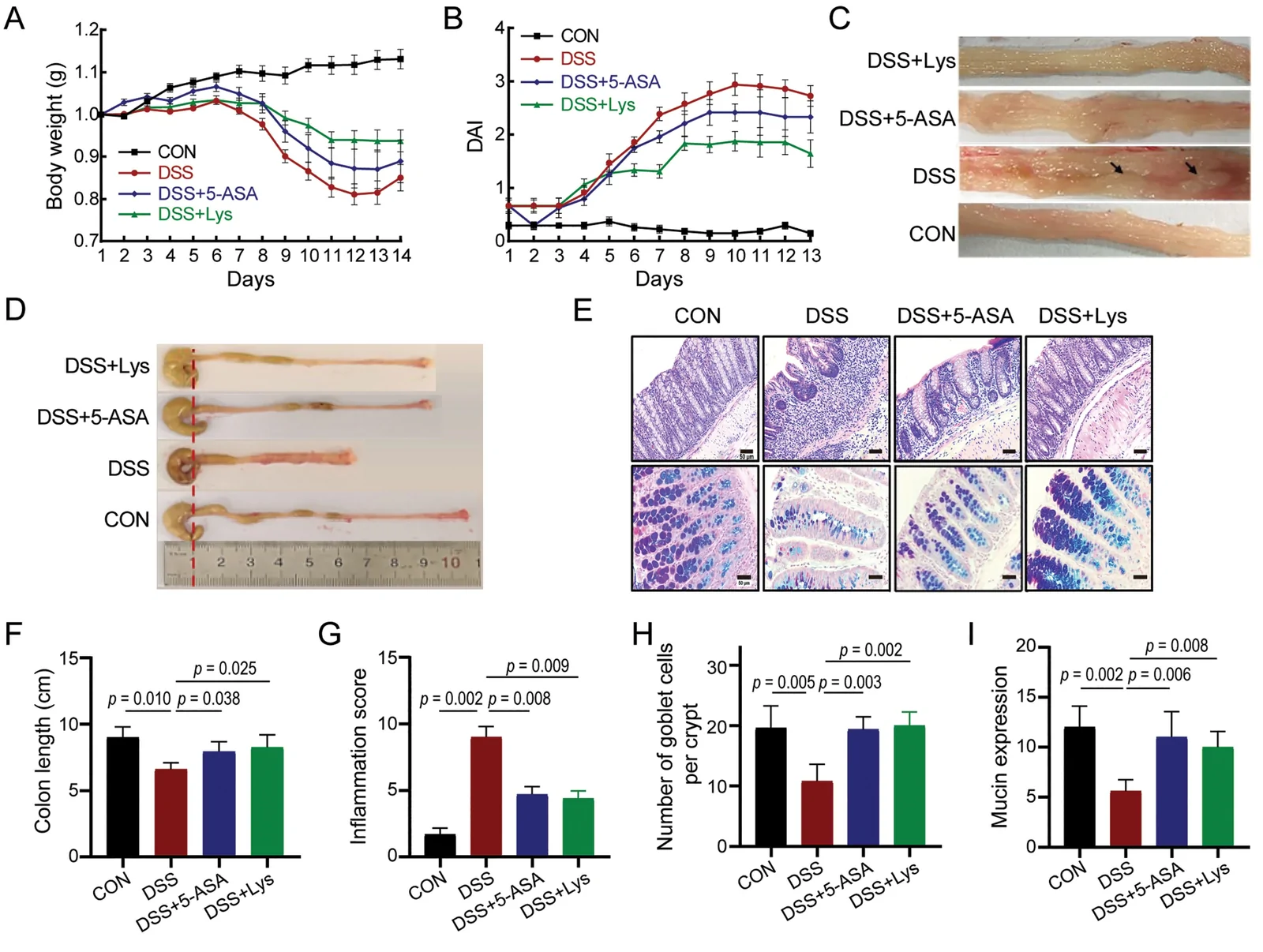
Lys relieves DSS-induced colonic pathological damage and mucous layer damage. (A) Weight change in four groups during the experiment, *n* = 15/group. (B) DAI change in four groups during the experiment, *n* = 15/group. (C) Representative photographs of colonic edema in all four groups. (D) Pictures of colon length of each group of mice. (E) Colon tissue of each group stained with H&E (upper panel) and AB-PAS (lower panel), scale bar = 50 μm. (F) Statistical analysis of colon length, *n* = 15/group. (G) Inflammation score estimated from H&E straining, *n* = 5/group. (H) Statistics of intestinal crypt goblet cells estimated from AB-PAS staining, *n* = 5/group. (I) Expression of colonic mucin estimated from AB-PAS staining, *n* = 5/group. One way ANOVA followed by Tukey’s post hoc test was used for statistical analysis. Data presented as mean ± SEM.

This study aimed to evaluate the therapeutic effects of Lys on DSS-induced colitis in mice. DSS is a high-molecular-weight sulfated polysaccharide commonly used to induce colitis, mimicking human inflammatory bowel diseases (IBD). It damages the intestinal epithelium, causing crypt damage, goblet cell loss, and infiltration of inflammatory cells in the submucosa, which are hallmarks of colitis. During the initial week of the experiment, no significant changes in body weight were observed across the experimental groups (Fig. 1A). However, starting from the second week, 400 mg/kg Lys treatment significantly mitigated the weight loss induced by DSS, showed comparable effect to the widely used clinical drug, 5-aminosalicylic acid (5-ASA) (Figs. 1A and S1). By the second week, clinical symptoms such as reduced food intake (Fig. S2) and slower movement appeared in the DSS group, and also the 5-ASA and Lys-treated groups (data not shown). Despite these symptoms, the disease activity index (DAI)—a standard measure of disease severity—was significantly lower in the Lys-treated group compared to the DSS group (Fig.1B). On the final day of the experiment, the DAI values for the groups were: 0.33 for the control group, 2.66 for the DSS group, 2.33 for the 5-ASA group, and 1.66 for the Lys group (Fig.1B). This indicated that the Lys group had a 1.6 times lower DAI compared to the DSS group and a 1.4 times lower DAI compared to the 5-ASA group, demonstrating the effectiveness of Lys in alleviating colitis symptoms.

Colon swelling and shortening are important indicators used to assess the severity of DSS-induced colitis. At the end of the experiment, colon measurements and morphological analysis revealed a significant swelling in the DSS group (Fig. 1C), along with a notable reduction in colon length (Fig. 1D and 1F). However, both the 5-ASA and Lys-treated groups showed alleviation of these effects (Fig. 1C, 1D, and 1F). The colon weight-to-length ratio, which is used to evaluate mucosal edema, was significantly higher in the DSS group, but Lys and 5-ASA treatments significantly reduced this increase (data not shown), showing no significant difference between the two treatments.

Histological examination and H&E-stained colon sections revealed that the control group exhibited intact colonic crypts and a well-preserved mucosal structure (Fig. 1E, upper panel). In contrast, the DSS group showed severe destruction of the mucosal layer, characterized by crypt deformation, loss of goblet cells in the upper mucosa, and substantial infiltration of inflammatory cells, such as neutrophils and monocytes, in the submucosa. Treatment with 5-ASA or Lys largely restored mucosal integrity, with fewer inflammatory cell infiltrations observed in the submucosa compared to the DSS group. The colonic inflammation scores, which assess the severity of colitis, confirmed that Lys treatment helped preserve the colonic mucosal barrier and protected the goblet cells and colonic glands (Fig. 1G).

To further assess the effect of Lys on mucosal damage, we employed Alcin-blue and periodic acid-Schiff (AB-PAS) staining techniques to visualize mucus proteins in the colon (Fig. 1E, lower panel). The number of goblet cells and the area of mucins were quantified (Fig. 1H and 1I). Consistent with the H&E staining results, DSS administration markedly reduced goblet cells, while treatment with 5-ASA or Lys significantly restored their numbers to baseline levels. Mucus proteins in the control group were widely and densely distributed, whereas DSS administration severely disrupted mucus protein production (Fig. 1I). Consequently, treatment with 5-ASA or Lys largely restored both the production and distribution of mucus proteins.

**Figure 2.**
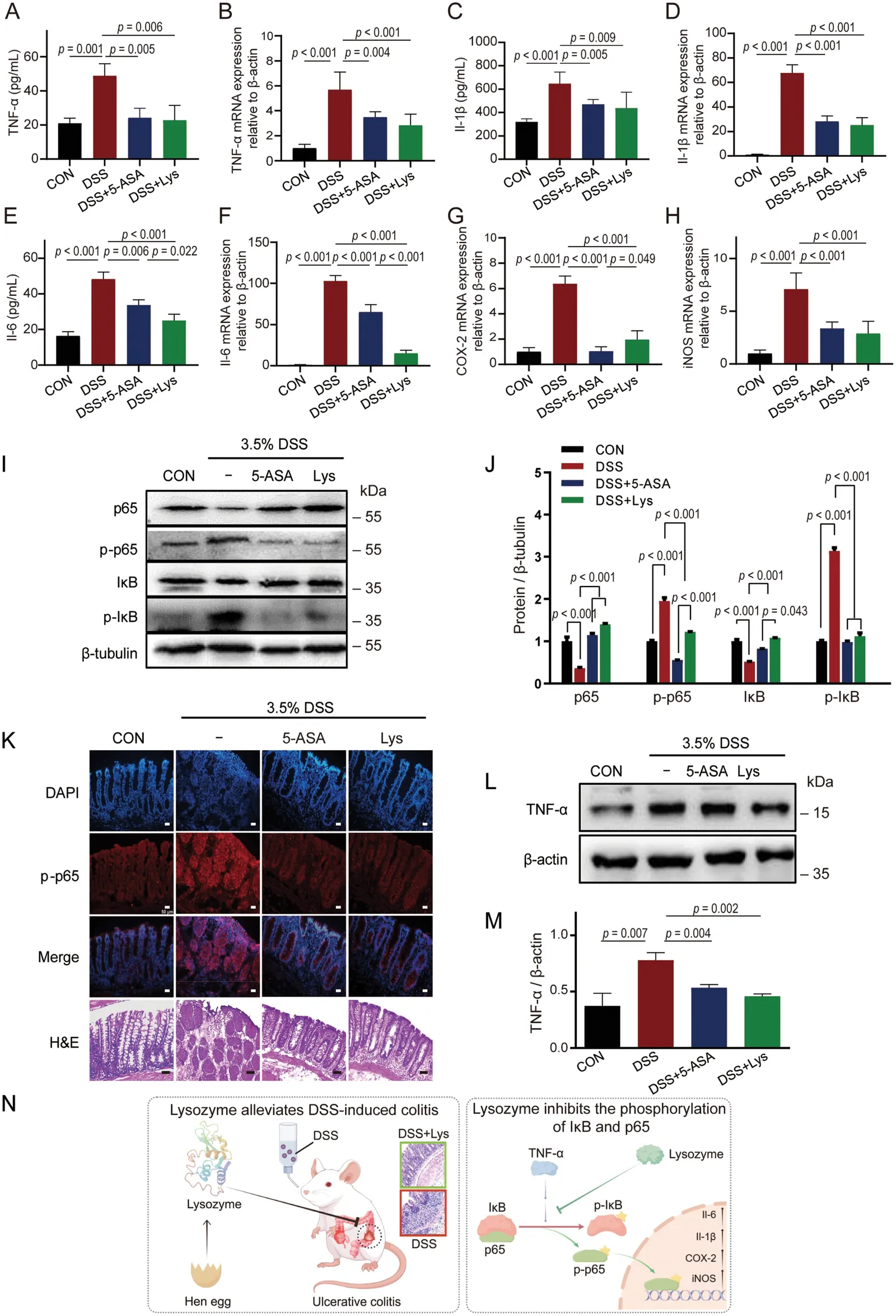
Effect of Lys on DSS-induced inflammatory response and NF-**Κ**B pathway activation. Serum levels of TNF-α (A), IL-1β (C), and IL-6 (E) in all four groups measured by ELISA, *n *= 5/group. mRNA expression levels of TNF-α (B), IL-1β (D), IL-6 (F), COX-2 (G) and iNOS (H) measured by RT-qPCR, *n *= 5/group. (I) Protein expression levels of total p65, p-p65, IκB, and p-IκB analyzed by Western blot. (J) Statistics of (I) analyzed by Image J, *n* = 3/group. (K) Expression and subcellular localization of p-p65 in colon visualized by immunofluorescence staining, scale bar = 50 μm. (L) TNF-α protein levels determined by Western blot analysis. (M) Statistics of (L) analyzed by Image J, *n* = 3/group. (N) Graphical summary. This cartoon was created with Figdraw. The hen egg lysozyme 3D structure was downloaded from PDB database (code: 1LYZ). One-way ANOVA followed by Tukey’s post hoc test was used for statistical analysis. Data presented as mean ± SEM.

The relationship between pro-inflammatory cytokines and colitis is well-documented in the literature [5, 10]. To explore whether Lys intervention could influence the expression of these cytokines following DSS administration, we analyzed both serum and colon samples. As shown in Fig. 2A–F, DSS administration significantly increased the expression levels of TNF-α, IL-1β, and IL-6. Both 5-ASA and Lys treatments significantly reduced the expression of TNF-α, IL-1β, and IL-6 compared to the DSS group. However, there were no significant differences in TNF-α and IL-1β levels between the 5-ASA and Lys treatment groups. Interestingly, Lys exhibited a more pronounced effect in reducing IL-6 levels compared to 5-ASA, as evidenced by lower IL-6 protein levels in the serum and decreased IL-6 mRNA expression in the colon.

COX-2 and iNOS are key enzymes involved in inflammation-related intestinal pathology. Stimulation by pro-inflammatory cytokines leads to elevated expression of COX-2 and iNOS in colon tissue. As shown in Fig. 2G–H, the mRNA expression levels of COX-2 and iNOS were significantly elevated in DSS-treated colon tissue compared to the control group. Following 2 weeks of 5-ASA and Lys treatment, the mRNA expression levels of both COX-2 and iNOS were significantly reduced.

NF-κB plays a central role in regulating inflammation, with its activation leading to the secretion of various pro-inflammatory cytokines. The overproduction of these inflammatory factors contributes to the progression of UC, resulting in persistent intestinal mucosal inflammation. NF-κB p50/p65 typically resides in the cytoplasm, sequestered by its inhibitor, IκB. Upon activation, NF-κB translocates to the nucleus, where it induces the expression of IκB, thereby inhibiting inflammation. TNF-α and other cytokines activate NF-κB through phosphorylation of p65 and IκB, leading to the translocation of phospho-p65 (p-p65) into the nucleus, where it regulates various target genes [5]. To investigate this pathway, we assessed the levels of p65, p-p65, IκB, and p-IκB in colonic tissues via Western blot analysis (Fig. 2I and 2J). We also examined the nuclear expression and localization of p-p65 using immunofluorescence in all four experimental groups (Fig. 2K). As shown in Fig. 2I, levels of p65 and IκB were reduced, while p-p65 and p-IκB were significantly elevated in the DSS group, indicating robust activation of the NF-κB signaling pathway. Figure 2K reveals that p-p65 levels were notably upregulated and translocated into the nucleus in the DSS group. Treatment with 5-ASA or Lys restored the levels of these proteins to baseline and reversed the translocation of p-p65 to the nucleus. DSS administration also led to elevated TNF-α expression, but both 5-ASA and Lys interventions significantly reduced TNF-α expression (Fig. 2L and 2M), with no significant differences observed between the 5-ASA and Lys treatment groups.

In conclusion, Lys from chicken egg white demonstrates considerable promise as a treatment for UC, particularly in alleviating symptoms and reducing inflammation in DSS-induced colitis (Fig. 2N). Its ability to restore mucosal integrity, regulate inflammatory cytokine levels, and modulate key inflammatory pathways underscores its potential as a therapeutic agent for UC and possibly other inflammatory disorders.

## Research limitations

The mouse model of DSS-induced colitis used in this study may not fully replicate the complexity of human IBD such as UC or Crohn’s disease (CD). While DSS-induced colitis is a widely used model, it primarily focuses on acute inflammation and does not account for the chronic nature of IBD in humans. Additionally, the study did not explore the long-term effects or potential side effects of Lys treatment, which are important for evaluating its clinical applicability. The research also lacked investigation into the optimal dosage and administration frequency of Lys, which could vary in human treatments.

## Supplementary Material

lnaf020_suppl_Supplementary_Figures_S1-S2
