# Supplementary material for: Lysozyme alleviates DSS-induced colitis by modulating pro-inflammatory factors and NF-κB activation
Source: Life Med. 2025 Jun 14;4(5):lnaf020. doi: 10.1093/lifemedi/lnaf020 (PMC12507011; doi:10.1093/lifemedi/lnaf020)
Supplement: lnaf020_suppl_Supplementary_Figures_S1-S2 [file lnaf020_suppl_supplementary_figures_s1-s2.docx]

**Supplementary Information for:**

Lysozyme Alleviates DSS-Induced Colitis by Modulating Pro-Inflammatory Factors and NF-κB Activation

Kai Wang^1,2,#,*^, Lan Guo^2,#^, Ting Wang^3,#^, Fengmin Shi^1^, Meihong Fu^1^, Guojun Li^1^, Songli Pan^1^, Xianying Cao^2,*^, Huanxiong Chen^4,*^

^1^Department of Spine Surgery, the First Affiliated Hospital of Hainan Medical University; International Center for Aging and Cancer; Hainan Academy of Medical Sciences, Hainan Medical University, Haikou 571199, China

^2^College of Food Science and Technology; State Key Laboratory of Marine Resources Utilization of South China Sea, Hainan University, Haikou 570228, China

^3^Shanxi Bethune Hospital; Shanxi Academy of Medical Sciences; Tongji Shanxi Hospital; Third Hospital of Shanxi Medical University, Taiyuan 030032, China

^4^Department of Spine Surgery, Hainan Province Clinical Medical Center, the Second Affiliated Hospital of Hainan Medical University, Hainan Academy of Medical Sciences, Hainan Medical University, Haikou 570311, China

^#^These authors contributed equally to this work.

^*^Correspondence: chenhuanxiong86@163.com (H.C.), [cxying_02@163.com](mailto:cxying_02@163.com) (X.C.), kai.wang@muhn.edu.cn (K.W.)

1. **Materials and methods**

1.1 Reagents

Dextran sulfate sodium (DSS, molecular weight 36–50 kDa) was obtained from MP Biomedicals (Irvine, CA, USA). Lys from chicken egg white (70000 U/mg) was purchased from Sigma–Aldrich Co. (St. Louis, MO). Rabbit monoclonal antibodies p65, IκB, p-p65, TNF-α, β-Actin, β-tubulin, Cy3-conjugated, and Alex-conjugated secondary antibodies were procured from Abcam (Shanghai, China). Peroxidase-conjugated goat antibodies were obtained from Thermo Fisher (Shanghai, China). ELISA kits were acquired from Absin (Shanghai, China). RNA extraction kit, reverse transcription and cDNA synthesis kit, and RT-PCR kit were provided by Sangon Biotech (Shanghai, China). Hematoxylin–eosin (H&E) stain and fecal occult blood reagent were obtained from Baso Diagnostics, Inc. (Zhuhai, China). Periodic acid-Schiff staining kit was purchased from Solarbio (Beijing, China). DAPI Fluoromount-G® was purchased from Southern Biotechnology Associates, Inc. (UAB, USA). All other chemicals were purchased from Beyotime Biotechnology (Shanghai, China).

1.2 Animals

Male Balb/c mice (*n* = 60; 6–8 weeks, weighing 19–22 g; quality certification No: 43006700019625) were purchased from Changsha Tianqin Biotechnology Co., Ltd. (Changsha, Hunan, China). All mice were housed under SPF conditions with IVC independent air equipment facility, Suzhou Fengshi Laboratory Animal Equipment Co., Ltd. (Suzhou, Zhejiang, China) and were adapted to the environment for a week before initiation of the study. The mice were randomly allocated to five per cage to create a stable social structure and minimize fighting and aggression. They were housed under standard conditions (constant temperature of 23°C ± 2°C, indoor relative humidity of 55% ± 10%, 12 h dark/light cycles). All mice were provided abundant but quantified food and water, and were allowed to freely consume standard foods and fresh drinking water (provided by the Institute of Materia Medica in Hainan). All animal experiments were approved by the Committee for Animal Care and Use of Hainan Medical University (N° HYLL-2018-408) and conducted in accordance with an animal protocol.

1.3 Experimental design

The animals were randomly divided into four experimental groups (*n* = 15 for each group): control group (CON) with administration of 0.9% saline, model group with UC induced by providing drinking water ad libitum containing 3.5% (*w*/*v*) DSS to mice for a week (DSS), positive control group treated with 75 mg/kg/day 5-ASA p.o. plus 3.5% (*w*/*v*) DSS (DSS + 5-ASA), and dietary protein intervention group treated with preliminary defined 400 mg/kg/day Lys (Fig. S1) from chicken egg white p.o. plus 3.5% (*w*/*v*) DSS (DSS + Lys). We used 0.9% saline to dissolve 5-ASA and Lys. During the experimental period, the general state of the mice was recorded every day, including food intake, body weight, stool status, and fecal occult blood. All mice were sacrificed on Day-14. The entire colon (from caecum to rectum) was obtained, the length and weight were recorded, and then the colon was cleaned and segmented for subsequent experiments.

1.4 Observation of general state of mice

The food intake, body weight, and disease activity index (DAI) of the mice were generally observed. The daily food intake of each cage mouse was recorded. Animals were detected from day 2 to 14 for stool status, occult blood in stool, and body weight change. The DAI was measured as in **Table 1**, the DAI was calculated as follows: DAI = (stool score + stool blood score + body weight loss score)/3.

**Table 1**. Evaluation of the disease activity index (DAI)

| **score** | **Weight loss (%)** | **Stool status** | **Fecal occult blood** |
| --- | --- | --- | --- |
| 0 | <1% | Normal | No |
| 1 | 1%–5% | Moist / stick | + |
| 2 | 5%–10% | Soft | ++ |
| 3 | 10%–15% | Diarrhea | +++ |
| 4 | 15% | Diarrhea | Blood is visible to the naked eye |

1.5 Histopathology

Colon tissue was fixed in 10% formalin for a week. Histopathologic changes were observed by paraffin embedding, sectioned (5 μm) (Leica, Bensheim, Germany), and then stained with hematoxylin and eosin (H&E) and periodic acid-Schiff (PAS). Colonic histopathological scoring was completed by three experts from the Clinical School of Hainan Medical College. The inflammation score was evaluated based on **Table 2**, the inflammation score was calculated as follows: inflammation score = mucosal epithelial cell score + mucosa immune cell score + submucosal immune cell score.

**Table 2**. Score of hispathological colitis

| **Histological parameters** | | **Description** | **Score** |
| --- | --- | --- | --- |
| Mucosa | Epithelial cell | Prolonged epithelial cell or crypt | 1 |
|  |  | Destruction of barrie | 2 |
|  |  | Ulcer (30% < loss < 60%) | 3 |
|  |  | Ulcer (loss > 60%) | 4 |
|  | Immune cell | Mild infiltration | 1 |
|  |  | Moderate infiltration | 2 |
|  |  | Severe infiltration | 3 |
| Sub-mucosa | Immune cell | Mild infiltration | 1 |
|  |  | Moderate infiltration | 2 |
|  |  | Severe infiltration | 3 |

1.6 Enzyme-linked immunosorbent assay

Blood was collected from the animals’ fundus venous plexus, left standing at room temperature for 2 h, centrifuged to collect serum, and then stored at −80°C. The expression of TNF-α, IL-1β, and IL-6 was detected using the ELISA kit in accordance with the manufacturer’s instructions.

1.7 Western blot analysis

Colon segments (150 mg) were homogenized using the lysis buffer for Western blot protein extracts (Beyotime, Shanghai, China). Protein lysates (10 μg each) were separated by 8%–12% SDS-PAGE and then transferred onto PVDF membranes. Then, the membranes were blocked with 5% BSA for 90 min and incubated overnight with the primary antibodies (1:2000) at 4°C. After 2h incubation with horseradish peroxidase-conjugated secondary antibody (1:5000) at 37°C, immuno-detection bands were visualized by ECL solution (Tanon 5200 Multi, shanghai, Chain). The Image analysis system was used to quantify protein bands (ImageJ 8.0, USA).

1.8 RNA extraction and real-time RT-PCR

In accordance with the manufacturers’ instructions, the Trizol UNlQ-10 column type kit was used to extract total RNA, the Thermo Scientific cDNA synthesis kit was used to configure the RNA reverse transcription system, and the 2× SybrGreen qPCR Master Mix was used for real-time PCR detection using LightCycler480 II system (Roche, Rotkreuz, Switzerland). The primer sequence is shown in **Table 3**.

**Table 3**. Primer sequences used for qRT-PCR analysis

| **Genes** | **Forward primers** | **Reverse primers** |
| --- | --- | --- |
| *β-actin* | ACGATATCGCTGCGCTGGT | CGATGGAGGGGAATACAGCC |
| *IL-1β* | TACATCAGCACCTCACAAGCA | ATTAGAAACAGTCCAGCCCATAC |
| *IL-6* | TCTTGGGACTGATGCTGGTG | CATGTGTAATTAAGCCTCCGACT |
| *TNF-α* | AGGCTGCCCCGACTACGT | GACTTTCTCCTGGTATGAGATAGCAAA |
| *COX-2* | GATGACTGCCCAACTCCC | ATTCGCTCCTGGACCCAA |

1.9 Immunofluorescence analysis and fluorescence microscopic observations

Mice colon tissues were fixed with 4% paraformaldehyde and then embedded for cryostat sectioning. The frozen slices were incubated with primary antibodies against phospho-NF-κB p65 (Rabbit; 1:400; Abcam,UK) and TNF-α (mouse; 1:150; Abcam,UK) at 4°C overnight. The slides were washed for 5min three times and then incubated in the dark with Cy3-conjugated secondary antibodies (1:400; Thermo Fisher, USA) for 1 h at RT. Then, the secondary antibody solution was discarded, and the slides were washed for 5 min three times. The slices were fixed with a DAPI containing fluorescence anti-quenching agent. The slides were photographed under an Olympus laser scanning confocal microscope (Olympus Co., Ltd., Japan).

1.10 Statistical analysis

All data were analyzed using IBM SPSS Statistics 23 and are expressed as mean ± SME. Independent sample t test was used to compare two groups. One way ANOVA followed by Tukey’s post hoc test was used to compare more than two groups. Statistical significance was considered at *p* < 0.05.

1. **Data availability**

The data that support the findings of this study are available from the corresponding author upon reasonable request.

1. **Supplementary figures**

**
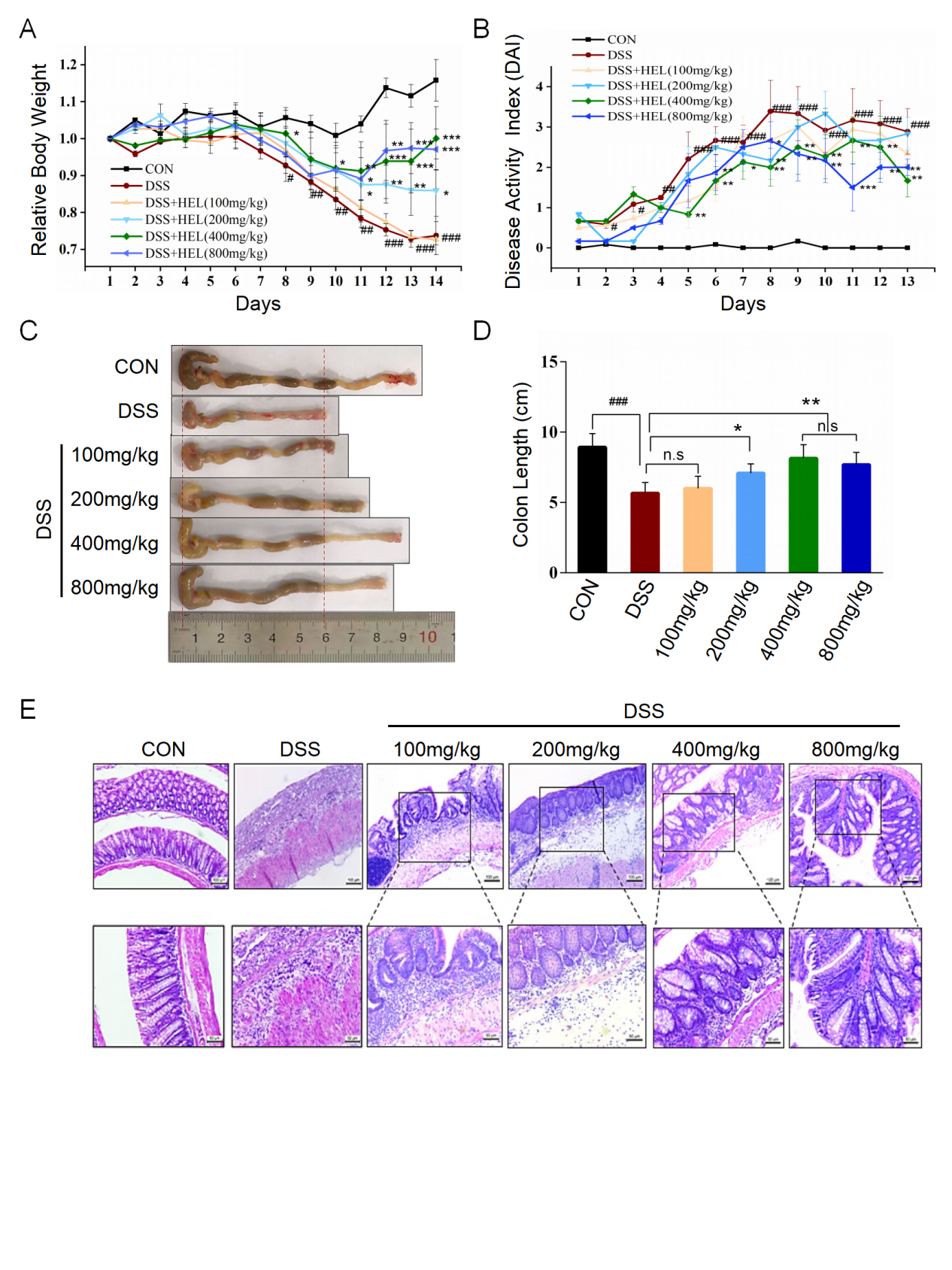
**

**Figure S1. Pilot experiment for determining the optimal lysozyme drug concentration**.

(A) Changes in body weight of experimental animals over time. (B) Changes in disease activity index (DAI) over time. (C) Representative colon images at the experimental endpoint. (D) Comparison of colon lengths across groups measured at the experimental endpoint after dissection. (E) Representative H&E-stained images of each group at the experimental endpoint, scale bar = 100 μm (upper panel) and 50 μm (lower panel). Statistical analysis was performed using one-way ANOVA followed by Tukey’s post hoc test. Data are presented as mean ± SEM. **p* < 0.05, ***p* < 0.01, ****p* < 0.001 indicate comparisons with the DSS group; ^#^*p* < 0.05, ^##^*p* < 0.01, ^###^*p* < 0.001 indicate comparisons with the CON group.


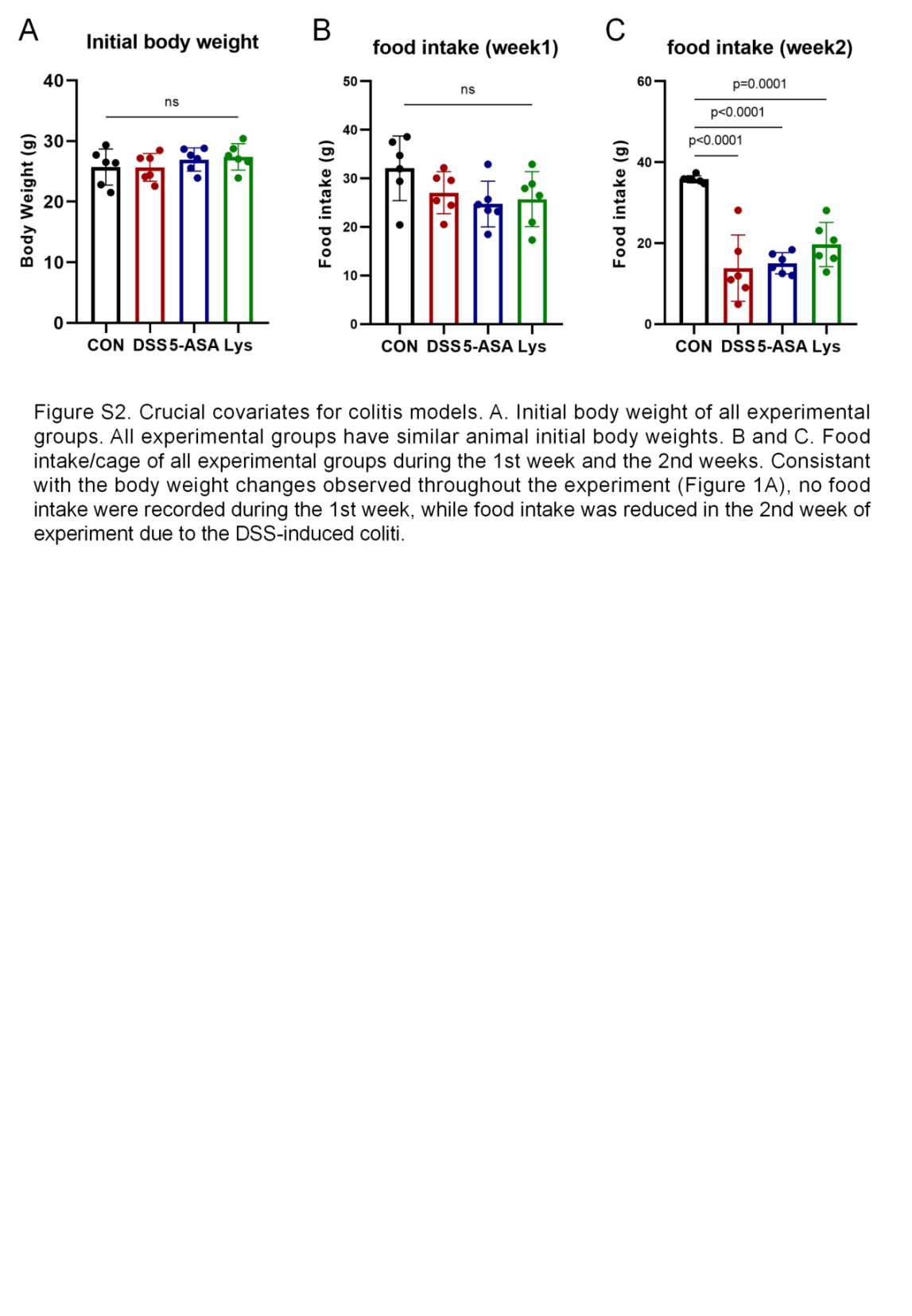


**Figure S2. Crucial co-variants for colitis models.**

(A) Initial body weight of all experimental groups. All experimental groups have similar animal initial body weights. (B, C) Food intake/cage of all experimental groups during the 1st week and the 2nd weeks. Consistent with the body weight changes observed throughout the experiment (Fig. 1A), no food intake were recorded during the 1st week, while food intake was reduced in the 2nd week of experiment due to the DSS-induced colitis.
